# MitoTimer probe reveals the impact of autophagy, fusion, and motility on subcellular distribution of young and old mitochondrial protein and on relative mitochondrial protein age

**DOI:** 10.4161/auto.26503

**Published:** 2013-09-27

**Authors:** Andrew W Ferree, Kyle Trudeau, Eden Zik, Ilan Y Benador, Gilad Twig, Roberta A Gottlieb, Orian S Shirihai

**Affiliations:** 1 Department of Medicine, Obesity and Nutrition Section; The Mitochondria Affinity Research Collaborative; Evans Biomedical Research Center; Boston University School of Medicine; Boston, MA USA; 2 Department of Medicine and the Dr. Pinchas Bornstein Talpiot Medical Leadership Program 2012; Sheba Medical Center; Tel-Hashomer, Israel; 3 Department of Molecular Cardiobiology; Heart Institute; Cedars-Sinai Medical Center; Los Angeles, CA USA; 4 Department of Clinical Biochemistry and Pharmacology; Faculty of Health Sciences; Ben-Gurion University of the Negev; Negev, Israel

**Keywords:** MitoTimer, aging, mitochondrial dynamics, autophagy, RHOT1

## Abstract

To study mitochondrial protein age dynamics, we targeted a time-sensitive fluorescent protein, MitoTimer, to the mitochondrial matrix. Mitochondrial age was revealed by the integrated portions of young (green) and old (red) MitoTimer protein. Mitochondrial protein age was dependent on turnover rates as pulsed synthesis, decreased import, or autophagic inhibition all increased the proportion of aged MitoTimer protein. Mitochondrial fusion promotes the distribution of young mitochondrial protein across the mitochondrial network as cells lacking essential fusion genes *Mfn1* and *Mfn2* displayed increased heterogeneity in mitochondrial protein age. Experiments in hippocampal neurons illustrate that the distribution of older and younger mitochondrial protein within the cell is determined by subcellular spatial organization and compartmentalization of mitochondria into neurites and soma. This effect was altered by overexpression of mitochondrial transport protein, RHOT1/MIRO1. Collectively our data show that distribution of young and old protein in the mitochondrial network is dependent on turnover, fusion, and transport.

## Introduction

As major producers of energy and oxidative stress, the maintenance of the mitochondrial network is critically important for cells. Mitochondrial quality control is especially crucial for long-lived cells with high energetic demands, such as neurons.^1^ Little is known regarding how newly synthesized mitochondrial proteins are distributed with existing, aged components.

Previous methodologies for studying mitochondrial aging have severe limitations including a lack of spatial resolution and live-cell applicability.^2^ We present a novel approach based on a time-sensitive fluorescent protein that is targeted to the mitochondrial matrix (MitoTimer). Emitted fluorescence of newly translated Timer is green and over time the emission shifts to red.^3^ We fused the fluorescent Timer to the mitochondrial targeting sequence of COX8A subunit, to form the MitoTimer construct. Our data identify mitochondrial turnover, fusion, and transport as factors involved in the equilibration of mitochondrial protein age. MitoTimer has utility as a read-out of mitochondrial maintenance with applicability across different tissues and disease contexts.

## Results

### Characterization and kinetics of MitoTimer

The fluorescent Timer protein fluoresces green post-synthesis. Within 24 h the emission spectra shifts to red fluorescence. We fused the fluorescent Timer to the mitochondrial targeting sequence of COX8A subunit, to form the MitoTimer construct. To control the timing of transcription of MitoTimer in cells, a doxycycline-inducible vector (pTRE-tight) was used to express MitoTimer. The resulting mitochondrial-targeted Timer localizes to mitochondria as assessed by co-localization with TOMM20 staining in MEF cells (Fig. S1).

Quantifying the time-dependent changes in green and red fluorescence of MitoTimer in MEF cells shows the initial green fluorescence 8 h after induction of transcription by doxycycline. After 24 h, MitoTimer is a mixture of green and red fluorescence, producing an overall yellow appearance. Over 48–72 h MitoTimer fluorescence becomes predominantly red (Fig. 1A and **C**) in transiently-transfected cells. Expressing MitoTimer in primary hippocampal neurons revealed similar time-dependent changes in green and red MitoTimer fluorescence (Fig. 1B).

**Figure:**
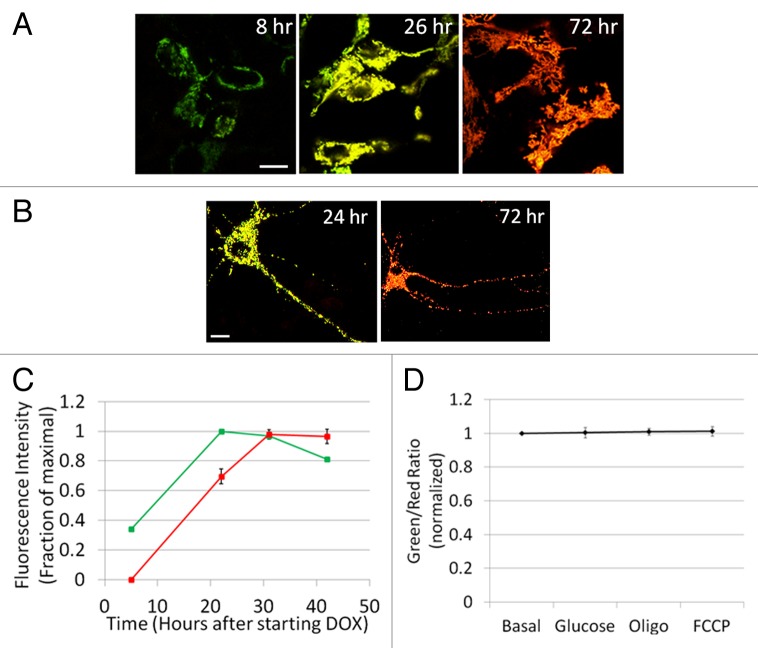
**Figure 1.** Localization and kinetics of MitoTimer. (**A**) MitoTimer was expressed in MEF cells by transfection followed by induction by doxycycline. Mitotimer showed mitochondrial localization and changed in color from green to red over time. Scale bar: 20 µm. (**B**) Primary hippocampal neurons were transfected and imaged at the different time points after doxycycline induction. MitoTimer showed similar localization to mitochondria and kinetics of green to red transition as in MEF cells. Scale bar: 20 µm. (**C**) Quantification of green and red fluorescence intensity over time after doxycycline (DOX) induction in MEF cells (n = 3). (**D**) MitoTimer green and red fluorescence (green/red ratio) was stable over a range of mitochondrial matrix pH conditions, achieved by acute exposure to 20 mM glucose, oligomycin (Oligo), or FCCP in INS1 cells stably-expressing MitoTimer (n = 10 cells).

Using a Leica TCS SP8 system, lambda-square scans were performed for sequential excitations produced by a white-light tunable laser in 10 nm steps and the maximal intensity recorded for the MitoTimer stably-expressed in a clonal β-cell line, INS1 cells. Figures S2 and S3 show excitation-emission spectra for green and red MitoTimer fluorescence, respectively. Although green and red fluorescence intensities of purified Timer protein were previously shown to be insensitive to pH changes within the physiological dynamics range,^3^ we performed additional confirmatory experiments to verify this issue in the context of the matrix-targeted Timer. To determine the effect of mitochondrial matrix pH changes on green and red MitoTimer fluorescence intensities, we altered matrix pH in INS1 cells that were stably-expressing MitoTimer. Matrix alkalinization was induced by acute treatment with high glucose (20 mM) and then with oligomycin (5 µM). These treatments were reported to bring matrix pH to pH~7.8 and pH~8, respectively.^4^,^5^ Matrix acidification was induced by treatment with 2 µM FCCP. FCCP was reported to bring matrix pH ≤ 7.^6^ The above modifying treatments did not affect the green-to-red fluorescence ratio in INS1 cells stably-expressing MitoTimer, consistent with previous reports of the pH-independent fluorescence of the Timer protein (Fig. 1D, Fig. S4A, n = 10 cells).^3^ In addition, MitoTimer red and green fluorescence were stable and unaffected by fixation, a treatment that completely dissipates the mitochondrial proton gradient (Fig. S4B, n = 3). Finally, the green-to-red color transition of MitoTimer was independent of protein expression, as average red and green MitoTimer fluorescence correlates similarly in INS1 cells with different overall expression (Fig. S4C). Thus, MitoTimer green-to-red fluorescence transition can be used as a read-out of relative mitochondrial protein age in different cell types.

### Turnover regulates mitochondrial network age

Rates of mitochondrial turnover depend on the balance between import of newly synthesized components and degradation of aged material. In addition to the time-dependent transition from green to red, MitoTimer age profile (overall color) will depend on rates of synthesis/import of new (green) protein and degradation of old (red) protein. To validate these associations, we quantified fluorescence in mitochondrial networks with pulsed MitoTimer synthesis, decreased protein import, or inhibited degradation by autophagy.

Limiting synthesis of new MitoTimer with a 4 h pulse-exposure to doxycycline altered the age profile relative to continuous 24 h doxycycline treatment. Pulsed expression shifted the age profile of MitoTimer in the mitochondrial network to increase the proportion of red relative to green fluorescence compared with continuous doxycycline (Fig. S5A compared with Fig. 1C). Plotting the red/green fluorescence ratio depicts the increased proportion of aged vs. new MitoTimer protein over time (Fig. 2A; *P* < 0.05, n = 4).

**Figure:**
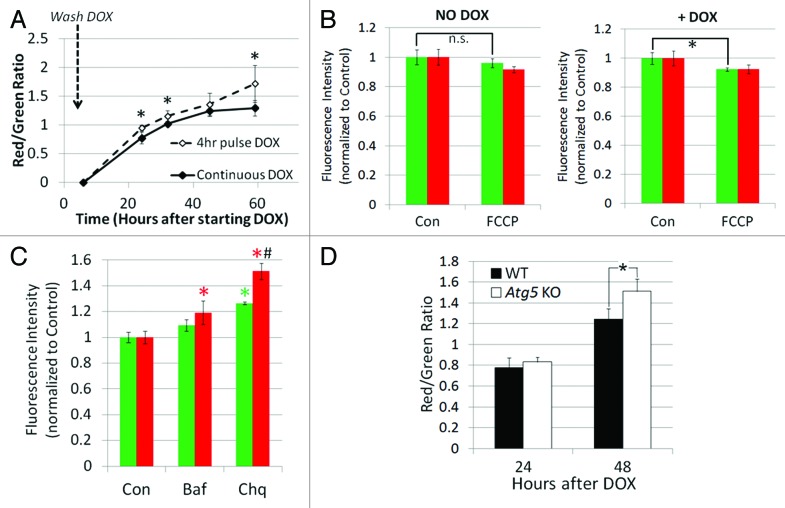
**Figure 2.** Relative abundance of young and aged MitoTimer protein is dependent on synthesis, import and autophagy. (**A**) As compared with continuous MitoTimer induction, limiting MitoTimer induction to 4 h of doxycycline pulse results in increased red/green ratio values (*P < 0.05, n = 4). (**B**) Effect of FCCP (10 µM, 3 h) on the abundance of green (green bar) and red (red bar) MitoTimer in the presence or absence of ongoing doxycycline-mediated MitoTimer synthesis. FCCP treatment in COS cells stably-expressing MitoTimer induced a significant decrease in green MitoTimer fluorescence (green bar) only in cells where active MitoTimer synthesis is ongoing (+DOX) (*P < 0.05, n.s. = non-significant, n = 3). (**C**) Inhibition of autophagy with bafilomycin (Baf; 100 nM for 16 h) or chloroquine (Chq; 30 µg/mL) in COS cells stably-expressing MitoTimer leads to significant accumulation of red fluorescence (red bar) relative to red fluorescence of control-untreated cells (red asterisk). Green fluorescence (green bar) in chloroquine-treated cells was significantly increased (green asterisk) compared with control-untreated cells. In chloroquine-treated cells, the proportional increase in red was significantly greater (pound sign) than the increase in green MitoTimer (*P < 0.05, ^#^*P* < 0.05, n = 3). (**D**) Cells deficient in autophagy (*Atg5* KO) show increased proportion of red MitoTimer after 48 h of expression, compared with wild-type (WT) MEFs (*P < 0.05, n = 3).

Similarly if import of new MitoTimer protein was limiting, we would expect a decrease in the amount of green MitoTimer fluorescence. We observed a modest yet significant decrease in MitoTimer green fluorescence after 3 h of FCCP (10 µM) treatment in COS cells stably-expressing MitoTimer (Fig. 2B; *P* < 0.05, n = 3). Importantly this decrease in green MitoTimer occurred only in the presence of doxycycline, which is required to allow transcription of new MitoTimer protein. Without doxycycline for the last 24 h before imaging, there was no significant change in green MitoTimer fluorescence after FCCP treatment (Fig. 2B). FCCP treatment caused a small, non-significant decrease in red MitoTimer fluorescence as well, which may be due to the fact that FCCP-induced depolarization of mitochondria also may promote mitochondrial turnover via autophagy. Overall, these data suggest that FCCP-induced depolarization prevents import of newly synthesized (green) MitoTimer protein into mitochondria.

To validate the capacity of Timer protein to measure the relative age of mitochondria in the cell, we inhibited mitochondrial turnover and measured green and red emissions. Turnover was inhibited by blockage of the autophagic degradation pathway in MEF cells transiently-expressing MitoTimer for 48 h. Inhibiting autophagy by preventing lysosome acidification with bafilomycin led to the accumulation of aged (red) material without significantly changing the amount of newly synthesized (green) protein (Fig. S5B; *P* < 0.05, n = 3). Similar results were seen with chloroquine treatment, which neutralizes lysosomal acidity. In order to address the effects of inhibiting mitochondrial turnover on MitoTimer age profile in steady-state condition, we generated stably-expressing MitoTimer COS cells via lentivirus infection. Treatment with bafilomycin or chloroquine similarly induced an accumulation of MitoTimer protein, with a greater proportion of red (old) protein accumulating (Fig. 2C, *P* < 0.05, n = 3). This effect was similar in cells that had not received doxycycline treatment for the last 24 h, which suggests the effect of bafilomycin and chloroquine was via inhibition of autophagy and independent of transcriptional effects (Fig. S5C).

Inhibition of autophagy was also achieved upstream of the lysosome with genetic knockout of *Atg5* in MEFs. After 48 h of continuous MitoTimer expression, mitochondrial networks in *Atg5* KO MEFs displayed a significantly increased average red fluorescence compared with wild-type MEFs (Fig. 2D; *P* < 0.05, n = 3), confirming that reduced protein turnover by autophagy leads to the accumulation of red (aged) MitoTimer. Collectively these findings are in agreement with the expected influence of mitochondrial turnover on accumulation of aged material within mitochondrial networks.

### Equilibration of protein age in the mitochondrial network requires fusion

We next examined whether mitochondrial dynamics influences the equilibration of protein age among individual mitochondria within the network. While individual mitochondria within the cell may import and degrade proteins at different rates, their involvement in continuous cycles of fusion and fission events may counter subcellular heterogeneity by allowing for the equilibration of matrix protein content. We rationalized that the ability to become engaged in fusion events may influence the level of heterogeneity in protein age among mitochondria in the cell.

Double-knockout MEFs lacking both *Mfn1* and *Mfn2* (*Mfn1/2* KO) show significant subcellular heterogeneity of MitoTimer age profiles compared with wild-type MEFs (Fig. 3B vs. Fig 3A).^7^ This MitoTimer heterogeneity was apparent in *Mfn1/2* KO MEFs transiently-transfected with and expressing MitoTimer for 24 h (Fig. S6A and S6B), but was even more striking in stably-expressing cells expressing MitoTimer for > 5 d (Fig. 3A and B). Assessing the average green and red fluorescence intensity of individual mitochondria (normalized to the average intensity for the cell) reveals the heterogeneity of MitoTimer age profiles in *Mfn1/2* KO cells compared with wild-type (Fig. 3C). While a majority of the mitochondria in *Mfn1/2* KO cells show similar relationships between green and red fluorescence as in wild-type, we observe a large number of appreciably redder or greener mitochondria in *Mfn1/2* KO cells. Plotting the red/green ratio of individual mitochondria vs. their size shows that the smallest units have the greatest heterogeneity of MitoTimer age profile in *Mfn1/2* KO MEFs (Fig. 3D; Fig. S6C). Overall, MitoTimer heterogeneity in *Mfn1/2* KO MEFs is significantly increased compared with wild-type MEFs (Fig. 3E; Fig. S6D; *P* < 0.01, n = 3). We also determined that inhibition of mitochondrial fusion by *Opa1* knockdown in INS1 cells resulted in increased subcellular heterogeneity of MitoTimer age profile (Fig. 3F and G; *P* < 0.05, n = 17 cells for control, 27 cells for *Opa1* knockdown from two independent infection procedures). Overall these data indicate the importance of mitochondrial fusion in equilibrating the levels of new and old protein within the network.

**Figure:**
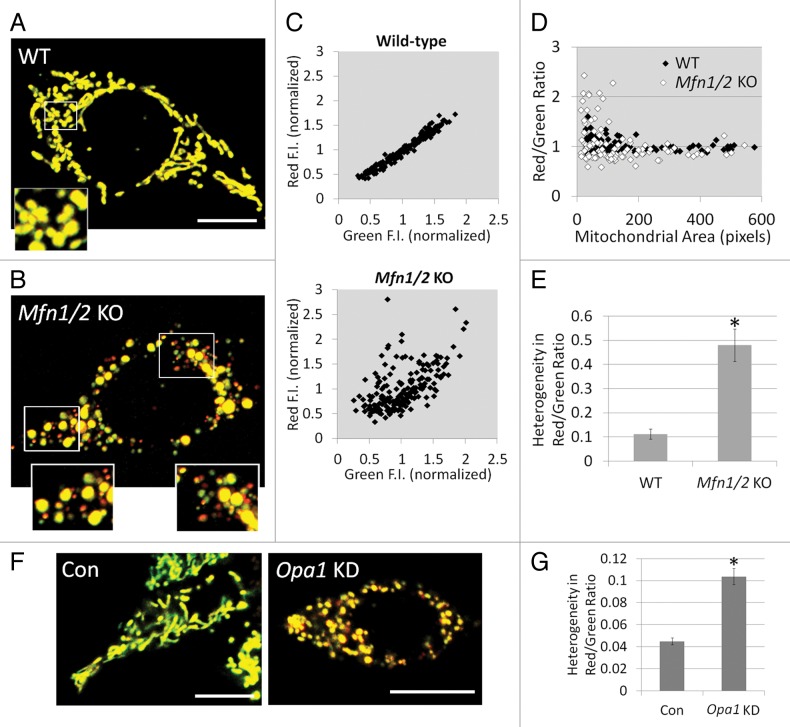
**Figure 3.** Fusion within the mitochondrial network mixes old and new proteins thus equilibrating MitoTimer age profiles. (**A**) Wild-type (WT) MEFs stably-expressing MitoTimer show relatively uniform yellow fluorescence in the mitochondrial network, indicating even distribution of old and new protein among mitochondria. Scale bar: 10 µm. (**B**) Mitochondrial fusion-deficient (*Mfn1/2* KO) MEFs stably-expressing MitoTimer display heterogeneity of MitoTimer red/green ratio among individual mitochondria compared with wild-type MEFs. Insets show high magnification of white boxes where small red and green mitochondria are apparent. (**C**) Graph depicts average green and red fluorescence intensity of individual mitochondria (normalized to the average green and red for the cell) in wild-type and *Mfn1/2* KO cells. MitoTimer age profiles vary widely in *Mfn1/2* KO cells (mitochondria from 4 representative cells). (**D**) Plotting the red/green ratio vs. area of individual mitochondria (mitochondria from 2 representative cells) shows the smaller mitochondria tend to display the greatest deviation from the average red/green ratio values in *Mfn1/2* KO MEFs (white diamonds) compared with wild-type MEFs (WT, black diamonds). (**E**) Subcellular heterogeneity in MitoTimer age (standard deviation of red/green ratio for all mitochondria in the cell) is increased in *Mfn1/2* KO MEFs compared with wild-type (WT) (*P < 0.01, n = 3). (**F**) INS1 stably-expressing MitoTimer and infected with *Opa1* knockdown lentivirus for 7 d showed intracellular heterogeneity of MitoTimer age profile compared with control-infected (Con) cells. Scale bars: 10 µm. (**G**) Subcellular heterogeneity in MitoTimer age is increased in *Opa1* knockdown (KD) INS1 cells compared with control (*P < 0.05, n = 17 cells for Control, 27 cells for *Opa1* KD from two independent infection procedures).

### Aged mitochondrial protein predominates in hippocampal neurites and is influenced by transport

We next turned to primary hippocampal neurons to examine MitoTimer age profiles in a cellular system with complex morphology and large spatial dimensions. As such, the neuron is a unique model in which one can study the impact of separate cellular compartments with disparate movement, fusion and biogenesis kinetics on the subcellular distribution of young and old protein.

MitoTimer expressed in primary hippocampal neurons for 24 h displays an overlap of green and red fluorescence in the soma producing an overall yellow appearance. In contrast, mitochondria in distal regions of neurites appeared more orange or red in fluorescence compared with the soma (Fig. 4A). This effect is illustrated with ratiometric images of the green/red fluorescence ratio (Fig. 4B). Equilibration of young and old protein across the neurites is limited by distance as aged MitoTimer predominates in distal neurite regions, shown by plotting the green/red ratio vs. distance from the soma for mitochondria in individual neurites (Fig. 4C). This analysis typically produced an inverse relationship characterized by a negative slope of the best-fit line (Fig. 4D, control group). The majority of neurites analyzed showed a negative slope while the rest show a slightly positive slope, indicating that neurons show heterogeneity of mitochondrial protein age in the neurites vs. the soma.

**Figure:**
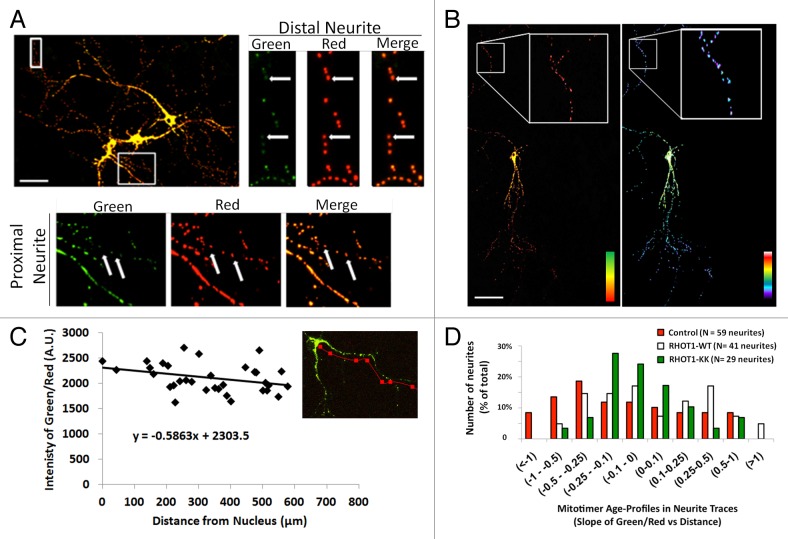
**Figure 4.** Aged MitoTimer is increased in hippocampal neurites and is regulated by mitochondrial motility. (**A**) Primary hippocampal neurons after 24 h of MitoTimer expression with mitochondria displaying overlap of green and red fluorescence. On the right, a high magnification of distal neurites is shown. Mitochondria with relatively little green fluorescence in neurites are designated with white arrows. Scale bar: 50 µm. At the bottom is a high magnification of proximal neurite. Note that proximal neurites have a relatively lower red/green ratio, indicative of younger MitoTimer protein. (**B**) Ratiometric (green/red) image of hippocampal neuron illustrating the predominance of aged MitoTimer in distal neurites. Scale bar: 100 µm. (**C**) Example of neurite age vs. distance trace analysis. All mitochondria within a single neurite were plotted based on green/red ratio intensity and distance from the nucleus. (**D**) Graph showing the distribution of slopes derived from the neurites age vs. distance trace analysis. The influence of mitochondrial motility is demonstrated by comparing baseline transport (Control) and enhanced transport (RHOT1-WT and RHOT1-KK) (ANOVA, *P* = 0.01 with Control vs. RHOT1-WT, *P* < 0.05).

Given that transport greatly influences subcellular spatial distribution of mitochondria, we chose to investigate the role of mitochondrial motility in the age equilibration pattern of MitoTimer in neurons. To increase mitochondrial transport, we co-expressed MitoTimer with RHOT1 wild-type (RHOT1-WT) or a hyperactive, calcium-insensitive mutant of RHOT1 (RHOT1-KK). RHOT1 is a motor adaptor protein that selectively facilitates mitochondrial movement along microtubules.^8^,^9^ Expressing the RHOT1 constructs altered the age distribution of MitoTimer in neurites (Fig. 4D; ANOVA, *P* = 0.01). Constitutively active RHOT1-KK increased homogeneity of the age profile between neurites and soma (distribution shifted toward zero). Overexpressing RHOT1-WT significantly reversed the normal distribution of aged material between the neurites and soma (distribution shifted to the positive, *P* < 0.05 vs. control). These data illustrate the importance of mitochondrial motility in equilibrating protein age between the soma and neurites.

To better understand the connection between spatial location and age heterogeneity, we performed time-lapse imaging to assess the dynamics of green and red MitoTimer in mitochondria located in the soma or neurites. Over the 12 h imaging period (imaging started after 24 h of MitoTimer expression), red MitoTimer fluorescence intensity increases in the soma and neurites as MitoTimer in both compartments transitions to red (Fig. 5A–C). Overall green intensity decreases earlier and more rapidly in mitochondria located in neurites compared with soma over the 12 h time-course (Fig. 5B and C; n = 12 neurons). This may reflect differences in rates of import of new (green) MitoTimer needed to balance the protein transitioning to red fluorescence. Thus, influx of new MitoTimer protein may be comparatively less for mitochondria located within neurites relative to those in the soma. In summary, these data suggest that fusion and transport are insufficient to prevent disequilibrium and heterogeneity in the age of the mitochondrial network located outside the neuron soma.

**Figure:**
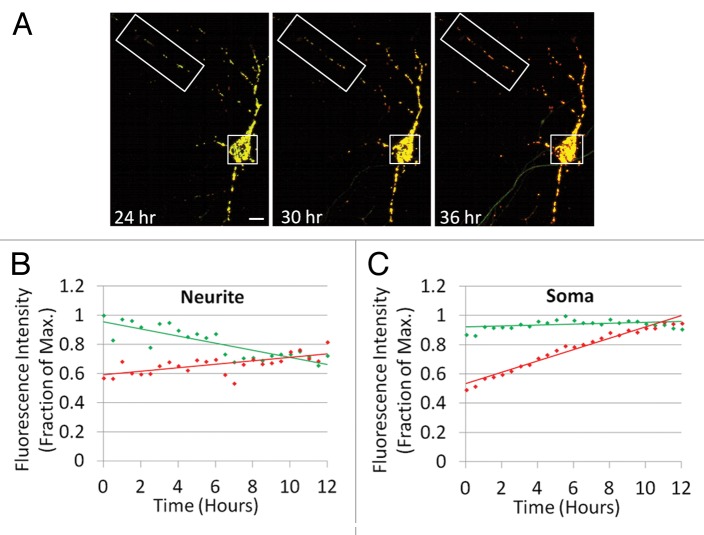
**Figure 5.** Kinetics of MitoTimer green-to-red color transition differs between neurites and soma. (**A**) Example of neuron in the live-cell imaging experiments used for the comparison of MitoTimer fluorescence kinetics in the neurites (**B**) vs. the soma (**C**). Graphs depict changes in MitoTimer green and red fluorescence over time for a representative neuron (n = 12 neurons). Scale bar: 10 µm.

## Discussion

Mechanisms for turnover of cellular constituents are central to the pathophysiology of age-related diseases. MitoTimer represents a novel tool to study mitochondrial turnover at cellular and subcellular levels (see illustration in Fig. 6). Control experiments with modulators of protein synthesis and autophagic degradation validate the age-dependent shift in fluorescence intensity as a read-out of turnover. We applied MitoTimer to test the influence of mitochondrial fusion on synchronization of mitochondrial protein age at a subcellular level. Equilibration of young and old protein across the network occurs in part through content mixing between mitochondria during complete fusion events.^10^ Heterogeneity in the MitoTimer age profile of individual mitochondria in cells lacking mitofusins reveals the necessity of fusion in equilibrating the age of proteins across the mitochondrial population.

**Figure:**
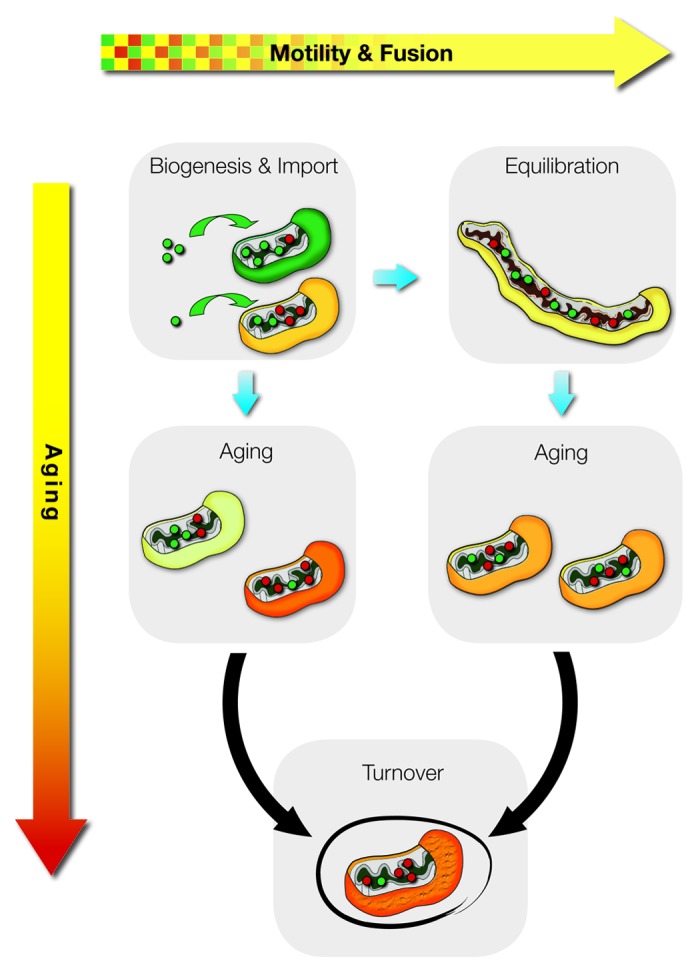
**Figure 6.** Illustration of mechanisms that can contribute to the distribution of aged (red) and young (green) MitoTimer protein. The vertical axis represents the effect of protein biogenesis and turnover on the overall cellular MitoTimer age. Mitotimer is formed green and turns to red with time. Since biogenesis generates only green MitoTimer while turnover removes both red and green, increased turnover will result in an increase in the portion of green mitochondria. The horizontal axis shows the processes that equilibrate young and aged MitoTimer across the cell. Reduced fusion activity either due to inhibition of fusion proteins, reduced motility or compartmentalization of a subgroup of mitochondria can all contribute to heterogeneous distribution of old and young mitochondrial proteins.

Hippocampal neurons also display dramatic heterogeneity among individual mitochondrial units, particularly in distal regions of neurites where aged MitoTimer predominates. This indicates that mitochondrial access to newly synthesized protein by either fusion events or by frequent visits to the soma is unlikely to contribute to biogenesis in mitochondria residing in the neurites. Support for this hypothesis is garnered from literature demonstrating that fusion rates in healthy neuronal processes are low,^11^,^12^ and that under normal conditions the majority of neurite mitochondria are stationary.^8^ Delivery of newly synthesized protein to isolated mitochondrial units in distal cell compartments, particularly of individual proteins with high turnover rates,^13^ may be supplemented by localized translation. Such a mechanism would likely involve targeting sequences not found on MitoTimer, as it is not a native mitochondrial protein. Given this possibility, it is noteworthy that variation in MitoTimer age profiles between soma and neurite can be influenced by microtubule transport activity. It has been demonstrated that mitochondrial motility is a significant determinant of fusion.^14^ Specifically, we demonstrate that enhancing motility by expression of wild-type RHOT1 shifts age profiles toward greater distribution of newly synthesized (green) MitoTimer. Recent studies also support this finding by showing that increased mitochondrial motility in neurons by RHOT1 overexpression increases fusion.^15^ RHOT1-WT, but not hyperactive RHOT1-KK, reversed the distribution of aged MitoTimer in neurites, suggesting calcium signaling that regulates RHOT1 activity may be crucial for proper distribution of contents throughout the mitochondrial network. Collectively these findings highlight the potential of therapies aimed at enhancing mitochondrial transport in treating conditions associated with impaired mitochondrial turnover in neurons, such as Parkinson disease.

Though MitoTimer represents a novel fluorescent probe to study mitochondrial maintenance, certain considerations have to be taken into account when assessing and interpreting results with this construct. Any changes to the readout of MitoTimer green, red, or red/green fluorescence intensity can only be interpreted when compared with a control condition at the same time point and imaged with identical acquisition settings. Importantly, changes to MitoTimer fluorescence cannot be interpreted as a specific alteration of mitochondrial biogenesis, degradation, dynamics, or motility per se. All these processes can potentially affect the age profile of MitoTimer in the cells (see illustration in Fig. 6); thus experimental conditions found to alter MitoTimer age profiles have to be further studied, with proper controls used, to determine which aspect of mitochondrial biology is actually affected. In addition, the read-out of increased red vs. green MitoTimer does not necessarily indicate increased damaged or dysfunction of mitochondria. Thus, MitoTimer can be used to assess relative changes to mitochondrial protein turnover and equilibration in different conditions or within the mitochondrial network.

Our work demonstrates how directly altering mitochondrial turnover, dynamics, or motility changes the age profile of MitoTimer. These conditions may represent experimental controls for further studies. Concurrent with this idea, MitoTimer is targeted to the mitochondrial matrix with a short mitochondrial-targeting sequence. Thus, it does not indicate the distribution or turnover of any specific mitochondrial protein, but rather acts as a marker for mitochondrial matrix protein content. It was recently shown that half-lives of mitochondrial proteins are very heterogeneous;^13^ thus it will be interesting for further studies to assess Timer kinetics and age profile when targeted to other mitochondrial compartments or tagged to specific mitochondrial proteins.

In summary, we report the characterization and applicability of a novel MitoTimer fluorescent probe, which can be used to study mitochondrial turnover and aging both on a cellular and subcellular level. Importantly we show that mitochondrial age as assessed by MitoTimer is dependent on mitochondrial turnover, fusion, and transport. Consequently, MitoTimer may be valuable as a fluorescent tool to investigate conditions where mitochondrial protein turnover is altered in different cells or pathologies.

## Materials and Methods

### Culture of cell lines

Matched *Atg5*^+/+^ (wild-type) and *Atg5*^−/−^ (*Atg5*-KO) mouse embryonic fibroblasts (MEFs) transfected with pEF321-T and an SV40 large T antigen expression vector were a generous gift from N Mizushima and were previously described.^16^ *Mfn1* and *Mfn2* double-knockout (*Mfn1/2* KO) MEFs were described previously.^17^ The three MEF cell lines and COS cells were grown in Dulbecco’s modified Eagle medium (DMEM) (Invitrogen, 10569-069) supplemented with 10% heat-inactivated fetal bovine serum (Invitrogen, 10437-028) and 1% penicillin and streptomycin (Invitrogen, 15140-122). INS1 832/13 cells were cultured in RPMI 1640 media (Invitrogen, 31800-089) supplemented with 10% fetal bovine serum (Invitrogen, 16000-044), 10 mM HEPES buffer (Cellgro, 25-060-Cl), 1 mM pyruvate (Cellgro, 25-000-Cl), 50 µM β-mercaptoethanol (Invitrogen, 21985), 1% penicillin, and streptomycin. Cells were plated on MatTek glass bottom dishes (MatTek, P35G-1.5-14-C) for confocal imaging and on 96-well plates (Corning, 3603) for imaging with Celigo Imaging Cell Cytometer.

### Isolation and culture of primary hippocampal neurons

Hippocampal tissue was harvested from timed pregnant (E14) CD1 mice and maintained in Neurobasal media (Invitrogen, 15140-122) with B27 supplement (Invitrogen, 15140-122) and Pen/Strep (Invitrogen, 17504044) with half-media changes every other day. Neurons were cultured on poly-d-lysine/laminin (Sigma, P6407-5MG) coated MatTek glass bottom dishes for confocal imaging. With the exception of the live-cell time-course studies, cells were fixed with 4% paraformaldehyde (Invitrogen, 15140-122) in phosphate buffered saline (PBS) prior to imaging.

### Transfection

MEFs were transfected using Lipofectamine LTX (Invitrogen, 15338-100). Neurons were transfected with calcium phosphate (Clontech, 631312) on the seventh day of in vitro culture (DIV7). Doxycycline (1 µg/mL; Invitrogen, 15140-122) was added to the media the day after transfection to induce MitoTimer expression.

### Creation of stable cell lines

COS, INS1, MEF wild-type, Atg5 KO, and *Mfn1/2* KO cell lines were infected with MitoTimer lentivirus and exposed to doxycycline to induce MitoTimer expression. After 72 h of doxycycline exposure, fluorescent cells were sorted into medium- and high-expressing populations with a BD FACSARIA II SORP cell sorter. Cells were then maintained without doxycycline for 2–3 passages before cells were plated and used for experiments. In order to conduct experiments with cells expressing steady-state levels of MitoTimer, stable cell lines were exposed to doxycycline for at least 5 d (doxycycline replaced daily) to obtain steady-state expression of MitoTimer before specific treatments or imaging was performed.

### *Opa1* knockdown

INS1 cells stably-expressing MitoTimer were infected with 2 different *Opa1*-shRNA lentiviruses targeted against different portions of the rat *Opa1* sequence. Both viruses were found effective at knocking down *Opa1* levels in INS1 cells resulting in a fragmented mitochondrial architecture. Cells were cultured for 6–7 d after infection with *Opa1*-shRNA lentiviruses before imaging.

### Immunofluorescence

To assess MitoTimer co-localization with mitochondrial Tomm20, immunofluorescence staining for Tomm20 was performed on MEFs transfected with MitoTimer. Briefly, transfected cells were fixed with 4% paraformaldehyde for 15 min. After washing in PBS, cells were incubated with 3% BSA (Millipore, 126575) in PBS with 0.2% Triton-X-100 (Sigma, T8787) for 30 min. Cells were then incubated overnight at 4 °C with a polyclonal rabbit anti-Tomm20 antibody (Abcam, 78547) diluted 1:200 in PBS containing 1% BSA. After PBS washes, cells were treated with anti-rabbit Alexa Fluor 405 (Invitrogen, A31566) secondary antibody diluted 1:200 in PBS containing 1% BSA. Cells were washed with PBS and imaged shortly after to visualize colocalization of MitoTimer with Tomm20 staining.

### Imaging

To quantify green and red fluorescence intensity per cell in MEFs and COS cells, cells were imaged using the Celigo Imaging Cell Cytometer (Brooks Life Science Systems). Green (483/32 excitation; 536/40 emission) and red (531/40 excitation; 629/53 emission) fluorescence channels were imaged for each well.

For subcellular imaging of MitoTimer expression, MEF, INS1 cells and primary hippocampal neurons were imaged by confocal microscope (Zeiss, LSM710) with a 63× oil immersion objective. MitoTimer was excited with 488 nm argon. Green fluorescence emission was collected in the 497–531 nm range while red fluorescence emission was collected in the 583–695 nm range. We observed that the red fluorescence will bleach faster than the green during multiple acquisitions. To control for possible bleaching during multiple acquisitions, cells were imaged multiple times with the laser settings used for experiments. Resulting images were then analyzed for the fluorescence intensity of red, green, and the red/green ratio in order to verify that successive imaging of the cells did not bleach either the green or red fluorescence. To observe individual mitochondria, z-stack images were acquired in series of six slices ranging in thickness from 0.5 to 0.8 µm. Tile imaging was performed to acquire the complete arborization of neurons, typically yielding 1–2 neurites per neuron used for analysis. Data for comparisons in neurites of control, RHOT1 WT, and RHOT1 KK groups were compiled from experiments done in replicate (3 < n < 5).

### Image analysis

Analysis parameters for images acquired by Celigo Imaging Cell Cytometer were optimized to identify individual MEF and COS cells based on fluorescence, and the average green and red fluorescence intensity per cell was determined at the different time points assessed. Specifically, the “Target 1+2 Merge” application setting in the Celigo software was used to determine the average green and red fluorescence for each cell selected based on the analysis parameters. Analysis settings were determined to identify fluorescent cells distinguishable from background fluorescence of cells not treated with doxycycline (no MitoTimer expression). Exposure and analysis settings were kept constant for each time-course experiment. Average fluorescence intensity per cell values were determined by the average integrated intensity per cell values in order to exclude error from background pixels included in identified cell regions. At least 1,000 cells were analyzed per well, with 3 well replicates per experiment.

To characterize MitoTimer fluorescence in individual mitochondria, a ratiometric image analysis approach was performed. An average of 8–12 cells was analyzed per experiment with experiments repeated at least 3 times (n = 3). Image processing and analysis was done with Metamorph software (Molecular Devices). Red and green images were used to generate a ratio (red/green or green/red) image. A maximum intensity projection of original z-stack images was generated to visualize the entire mitochondrial volume. Minimum thresholding for mitochondrial areas was set at 15% of the maximum pixel intensity for red images. Prior to analysis, all images were scanned to verify that all intensity measurements were below saturation; therefore an upper threshold was not necessary. The thresholded images were then binarized and single pixels removed. The identified mitochondrial areas were then applied to the green image, and a ratio image was generated by dividing the red by the green image (or green divided by red for green/red ratio images). Pseudocoloring was used to visualize the variation in red/green ratio among mitochondria. Fluorescence intensity (red/green) values of identified mitochondria were then determined to assess the differences in MitoTimer fluorescence among mitochondria in MEF cells or primary neurons, thus generating an age profile.

## Supplementary Material

Additional material

## Abbreviations:

MEF mouse embryonic fibroblast: PBS phosphate buffered saline, BSA bovine serum albumin, FCCP trifluorocarbonylcyanide phenylhydrazone, RHOT1-WT RHOT1 wild-type, RHOT1-KK RHOT1 calcium-insensitive mutant

## Notes

10.4161/auto.26503
