# Supplementary material for: MitoTimer probe reveals the impact of autophagy, fusion, and motility on subcellular distribution of young and old mitochondrial protein and on relative mitochondrial protein age
Source: Autophagy. 2013 Sep 27;9(11):1887–96. doi: 10.4161/auto.26503 (PMC4028338; doi:10.4161/auto.26503)
Supplement: Additional material [file auto-9-1887-s01.pdf]

Landes Bioscience

www.landesbioscience.com

LANDES BIOSCIENCE

Supplemental Material to:

Andrew W Ferree, Kyle Trudeau, Eden Zik, Ilan Y Benador, Roberta A Gottlieb, Orian S Shirihai

MitoTimer probe reveals the impact of autophagy, fusion, and motility on subcellular distribution of young and old mitochondrial protein and on relative mitochondrial protein age

Autophagy 2013; 9(11)

http://dx.doi.org/10.4161/auto.26503

www.landesbioscience.com/journals/autophagy/article/26503

Supplementary Figure 1

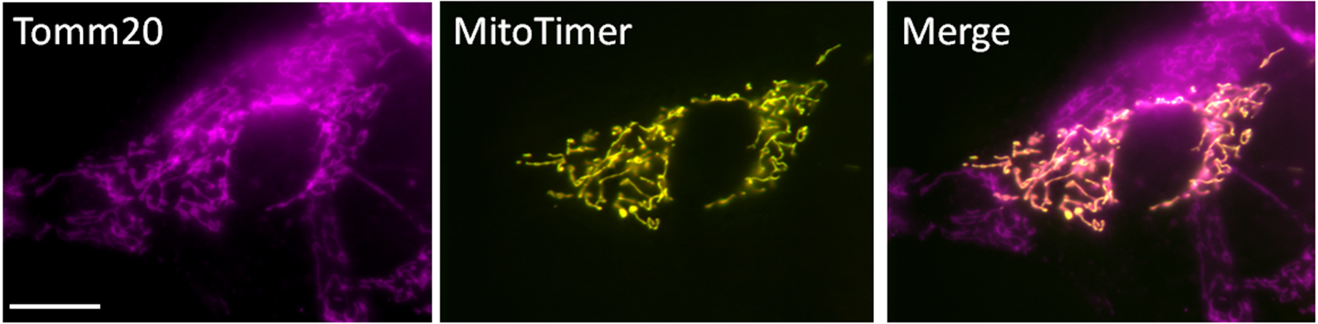

Supplementary Figure 2

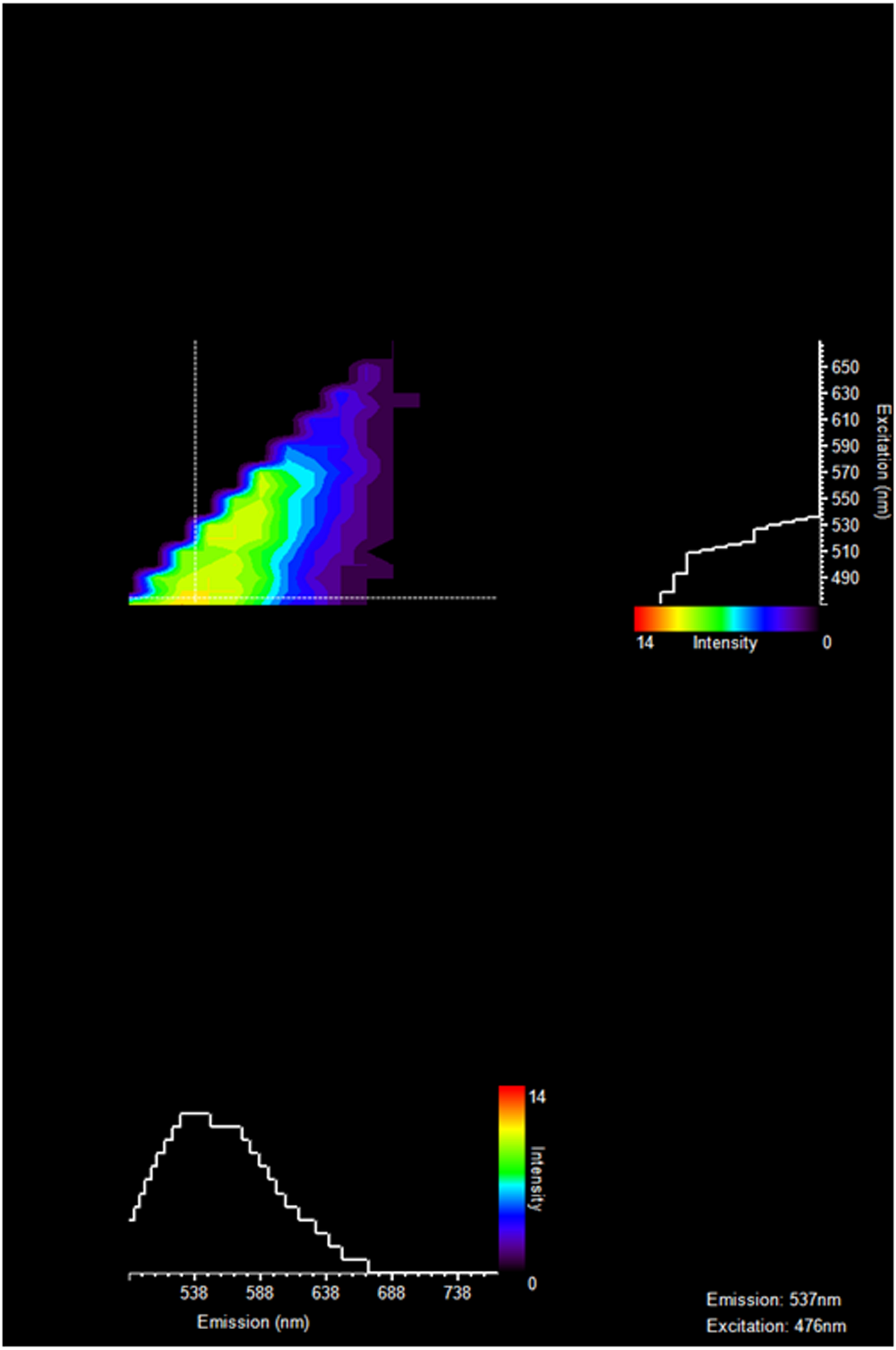

Supplementary Figure 3

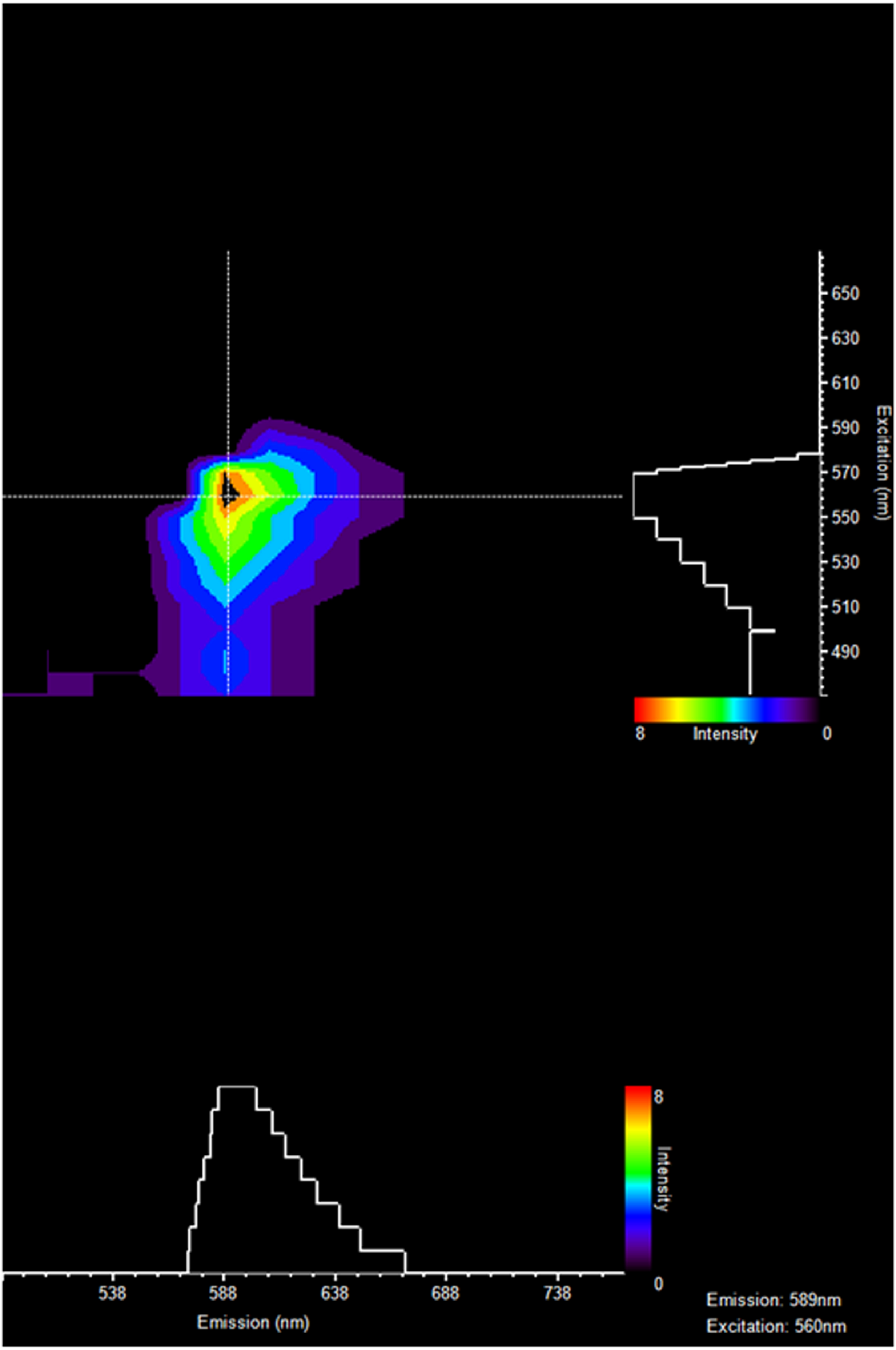

Supplementary Figure 4

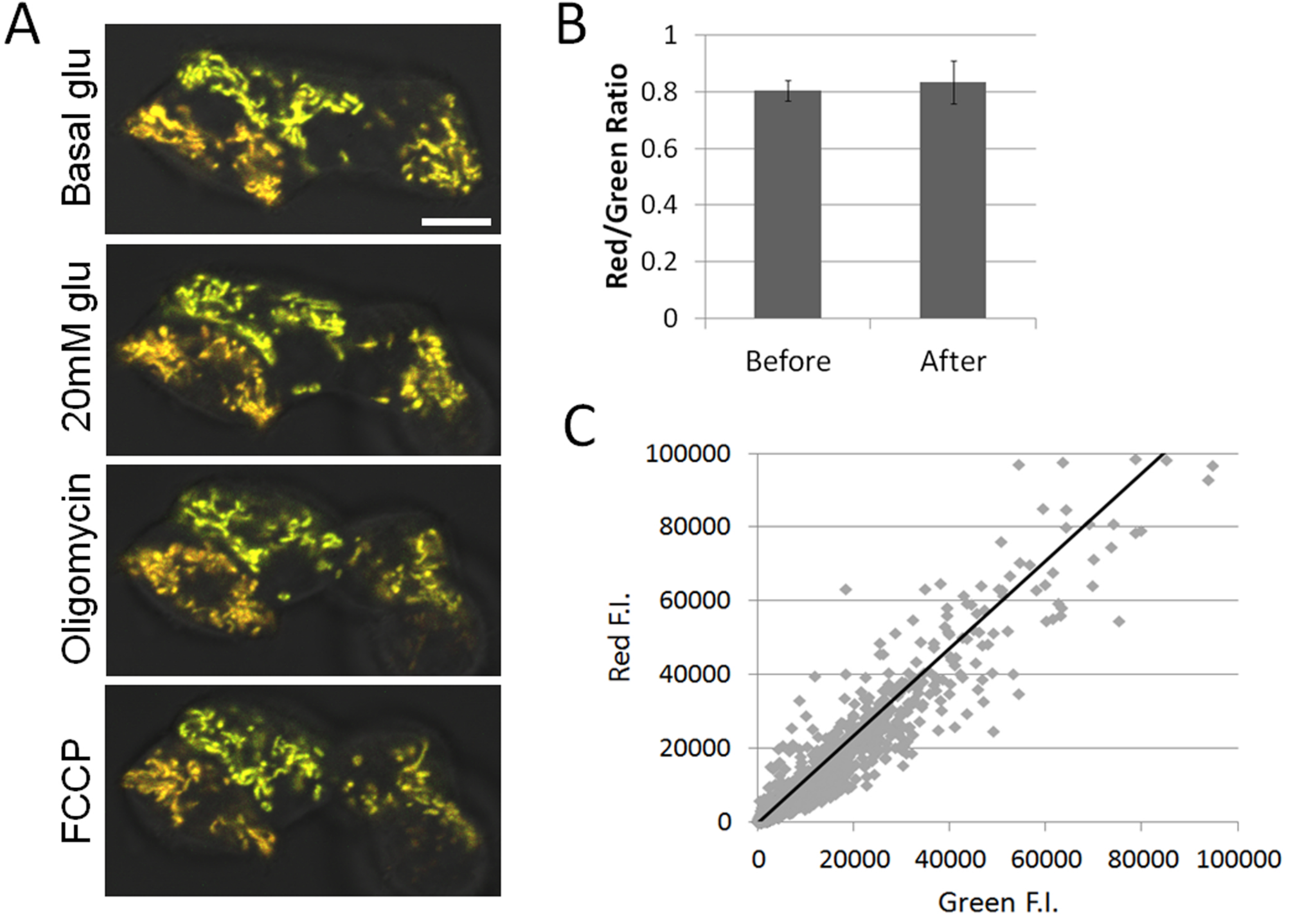

Supplementary Figure 5

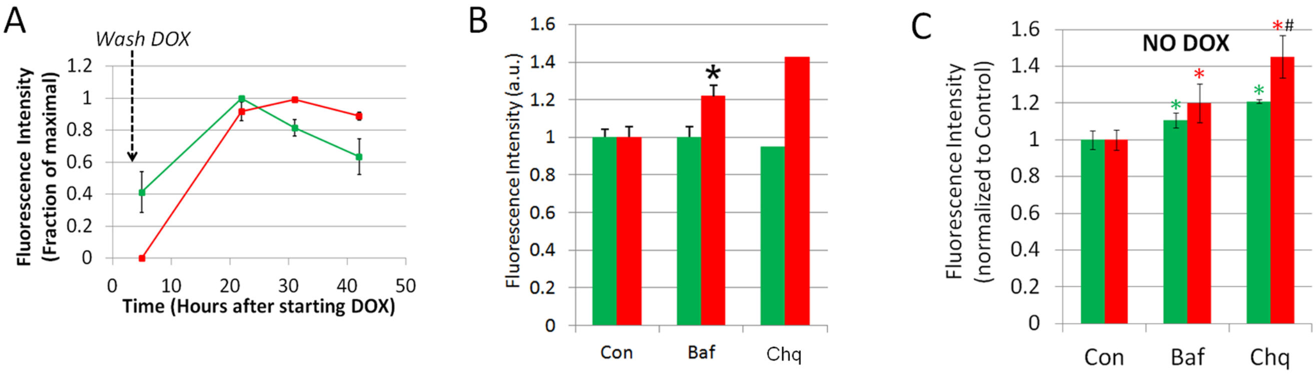

Supplementary Figure 6

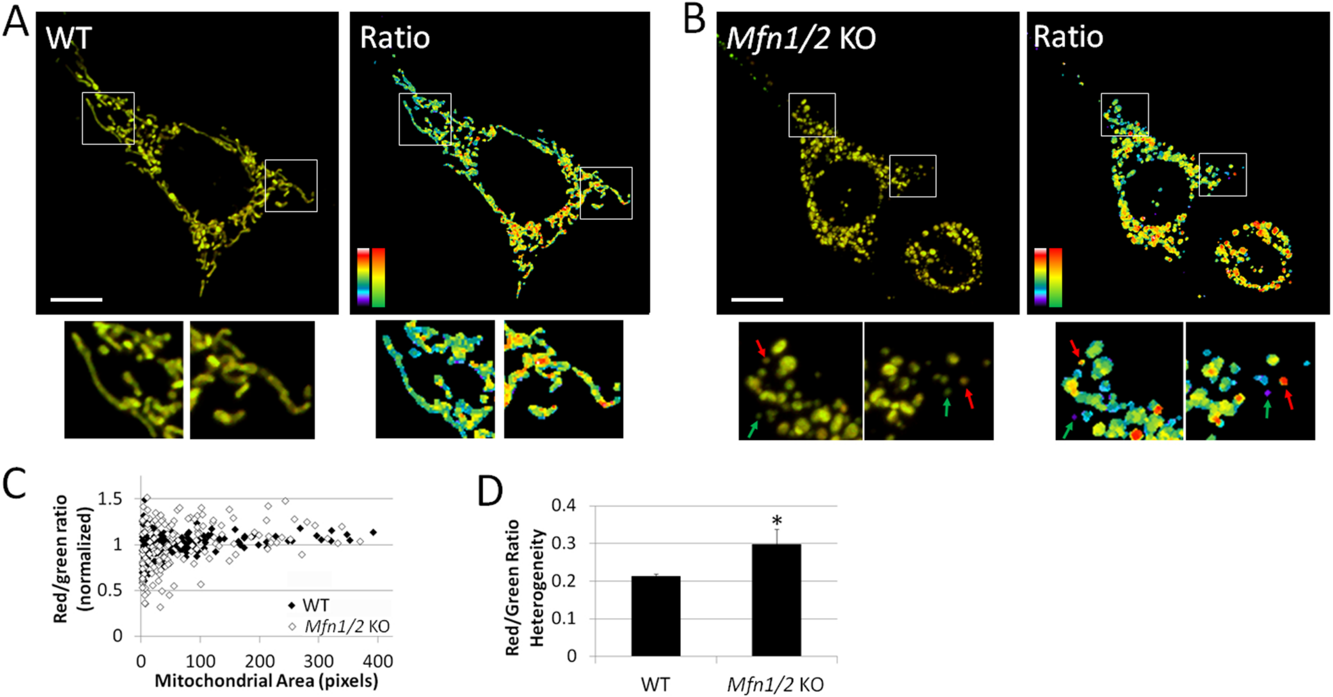

Figure S1. MitoTimer localizes to mitochondria in MEF cells. MitoTimer construct was transfected into MEF cells, and 24 h later cells were fixed and stained for TOMM20 conjugated with AlexaFluor405 secondary antibody (pseudo-colored magenta). MitoTimer is co-localized to mitochondria stained with TOMM20. Scale bar: 20 µm.

Figure S2. Excitation/emission spectra of MitoTimer green fluorescence. Lambda-square scans were performed for sequential excitations produced by a white-light tunable laser in 10 nm steps and the maximal intensity recorded.

Figure S3. Excitation/Emission spectra of MitoTimer red fluorescence. Lambda-square scans were performed for sequential excitations produced by a white-light tunable laser in 10 nm steps and the maximal intensity recorded.

Figure S4. MitoTimer green and red fluorescence is unaffected by pH changes or protein expression level. (**A**) Images of INS1 cells stably-expressing MitoTimer showing red and green fluorescence at basal glucose levels. MitoTimer fluorescence was not affected by acute exposure to high glucose (20 mM glu, 15 minutes), oligomycin (5 µM, 15 minutes), or FCCP (2 µM, 15 minutes). Scale bar: 10 µm. (**B**) COS cells stably-expressing MitoTimer were imaged and the red/green MitoTimer ratio was assessed. After fixation with 4% paraformaldehyde for 15 minutes followed by subsequent imaging, the red/green MitoTimer ratio was unchanged. (**C**) INS1 cells stably-expressing MitoTimer were analyzed by flow cytometry. Red and green MitoTimer fluorescence intensity (F.I.) increased with a linear relationship.

Figure S5. Limited synthesis or inhibition of autophagy increase the relative proportion of aged MitoTimer. (**A**) Pulsing MitoTimer production with 4 h of doxycycline (DOX) induction increases the proportion of red fluorescence over-time compared to continuous doxycycline induction (as shown in **Fig. 1D**). (**B**) Inhibition of autophagy with bafilomycin (Baf; 100 nM for 16 h) or chloroquine (Chq; 30 µg/mL) in MEF cells transiently expressing MitoTimer for 48 h leads to the

accumulation of red MitoTimer fluorescence (red bar) relative to green fluorescence (green bar) compared to control (Con) condition (*=p<0.05, n=3). (**C**) COS cells stably-expressing MitoTimer via continuous doxycycline (DOX) induction except for the last 24 h before imaging show significant accumulation of red fluorescence (red bar) relative to red fluorescence of control-untreated cells (red asterisk). Green fluorescence (green bar) in bafilomycin- and chloroquine-treated cells was also significantly increased (green asterisk) compared to control-untreated cells. In chloroquine-treated cells, the proportional increase in red was significantly greater (pound sign) than the increase in green MitoTimer (*$P < 0.05$, $^{\#}P < 0.05$, n = 3).

Figure S6. Lack of mitochondrial fusion in MEF or INS1 cells shows heterogeneity of MitoTimer age profile within the mitochondrial network. (**A**) Wild-type (WT) MEFs transiently expressing MitoTimer for 24 h show overall uniform yellow fluorescence of MitoTimer. Ratio image depicting red/green ratio of MitoTimer reveals relatively uniform age profiles among mitochondria in the cell. (**B**) Moderate heterogeneity of MitoTimer red/green ratio is seen in *Mfn1/2* KO MEFs expressing MitoTimer for 24 h. Green and red arrows indicate small mitochondria that are comparatively more green and red, respectively, than the majority of the mitochondrial population. Scale bar: 10 µm. (**C**) Graph shows that smaller mitochondrial units display greater heterogeneity of MitoTimer ratio in *Mfn1/2* KO MEFs (white diamonds) compared to wild-type MEFs (WT, black diamonds) expressing MitoTimer for 24 h. (**D**) MitoTimer heterogeneity (standard deviation of red/green ratio for all mitochondria in the cell) is increased in *Mfn1/2* KO MEFs compared to wild-type (WT) (*$P < 0.05$, n = 3).
